# Thermodynamic Analysis of Trisiloxane Surfactant Adsorption and Aggregation Processes

**DOI:** 10.3390/molecules25235669

**Published:** 2020-12-01

**Authors:** Joanna Karasiewicz, Joanna Krawczyk

**Affiliations:** 1 Chemistry and Technology of Silicon Compounds, Faculty of Chemistry, Adam Mickiewicz University in Poznań, Uniwersytetu Poznańskiego 8, 61-614 Poznań, Poland; 2 Department of Interfacial Phenomena, Institute of Chemical Sciences, Faculty of Chemistry, Maria Curie-Skłodowska University in Lublin, Maria Curie-Skłodowska Sq. 3, 20-031 Lublin, Poland

**Keywords:** organofunctional siloxanes, hydrosilylation, adsorption, micellization, thermodynamic properties

## Abstract

The trisiloxane polyether surfactant (3-[3-(hydroxy)(polyethoxy)propyl]-1,1,1,3,5,5,5 -heptamethyltrisiloxane) (TS-EO12) was successfully synthesized by a hydrosilylation reaction in the presence of Karstedt catalyst. The structural analysis of the surfactant was done by ^1^H-NMR, ^13^C-NMR, ^29^Si-NMR and FT-IR analysis. In addition the thermal stability of TS-EO12 was studied by the thermogravimetric measurements. On the one hand the surface properties of TS-EO12 at the water-air interface were investigated by surfactant aqueous solutions surface tension measurements carried out at 293 K, 303 K and 313 K, and on the other the aggregation properties were analyzed based on the solubilization properties of TS-EO12 aggregates at different temperatures. On the basis of the obtained thermodynamic parameters of adsorption and micellization of studied surfactant the temperature impact on its surface and volume properties were deduced. It was proved that the tendency of the studied surfactant molecules to adsorb at the water-air interface and to form micelles weakens with decreasing temperature. It was also concluded that the structure of the adsorption layer changes with temperature. Optical microscopy measurements were used for the TS-EO12 micelle morphology determination.

## 1. Introduction

Siloxanes are synthetic polymers whose skeleton is made up of alternately arranged silicon and oxygen atoms. Thanks to the presence of the Si–H bond, it is possible to attach all kinds of substituents, e.g., amino [1,2,3,4], epoxycarbo [5,6,7], aryl and methacrylic [8,9] or polyether [4,10,11] groups. Modification of polysiloxanes with organic groups provides a wide range of hybrid materials combining the properties of polysiloxanes (thermal resistance, oxidation resistance, gas permeability, low surface tension, excellent dielectric properties, physiological neutrality and moisture resistance) with those of organic functional groups. The hybrid materials obtained in this way may show a wide range of diverse properties meeting the needs of different branches of industry [12,13,14]. The most effective and widely used method for the introduction of different functional groups, for example polyether ones, to the polysiloxane chain is the addition to the Si–H bond in unsaturated compounds containing a desired functional group, so the process of hydrosilylation. The precursor containing the Si–H bond can be a siloxane polymer, cyclic siloxane oligomer and monomeric silane containing Si–H bond and two substituents prone to hydrolysis at the silicon atom. These reactions are usually catalyzed by platinum compounds [15,16,17,18]. Often encountered in literature is also the method for the synthesis of silicon polyethers by the reaction of hydrothiolation [19,20,21,22,23].

Modified siloxanes composed of the hydrophobic and hydrophilic moieties within the same molecule (amphiphilic nature) reduce the surface tension and exhibit a tendency to association and self-assembly. Thus they can be applied as surfactants [24,25,26]. Siloxane surfactants consist of a methylated siloxane hydrophobic group coupled to one or more polar groups. Functional groups can be attached through either a Si–C or a Si–O–C linkage.

The most popular hydrophilic groups present in siloxane surfactants are the non-ionic polyoxyalkylene, polyoxyethylene or copolymer of polyoxyethylene and polyoxypropylene. However, it is worth noting that a polyoxypropylene group is hydrophobic and contributes to the hydrophobicity of the surfactant rather than to its hydrophilicity. In addition, hydrophilic, higher alkyl, and fluorocarbon groups as well as a combination of these groups can be attached to the siloxane backbone to make materials that are surface active towards a variety of surfaces and interfaces. As siloxane surfactants show surface and interface activity, low toxicity and unique association behavior they find a variety of applications in the areas in which other types of surfactants are relatively ineffective. Another attractive feature of siloxane surfactants is a variety of their synthetic routes and the consequent diversity of their possible structures [27]. It is interesting that trisiloxane surfactants consisting of a polyoxyethylene polar group of four to eight units show the unique property of facilitating the spreading of water on very hydrophobic hydrocarbon surfaces such as polyethylene and paraffin [28,29]. As the special wetting properties of these surfactants are strictly connected with their unique structure, the ability to lower the liquid-air surface tension to extremely low values, fast kinetics of adsorption at different interfaces, high affinity for low energy surfaces as well as specific orientation and structure of their adsorbed molecules it is so important to establish these properties in different conditions [30].

The unique structure of siloxane surfactants results from the relatively flexible Si–O–Si bond which enables them to adopt a variety of configurations. It occurs that the molecular weight affects the relevant properties of such surfactants. However, it should be remembered that the increase in the molecular weight can be connected with the hydrophobic as well as hydrophilic part of surfactant. Compared with hydrocarbon surfactants, trisiloxane ones have considerably higher surface activity attributed to the trimethylsiloxy group on the Si−O−Si chain. Because of their unique molecular structures, they can absorb at the air/solution interface an “umbrella-like’’ configuration closely packed with the −CH_3_ groups and exposed to the lower surface tension values (about 21 mN/m). Gentle et al. [24] studied a series of polyether based trisiloxanes with different ethylene oxide (EO) chain lengths and proved that the critical micelle concentration (CMC) and surface tension at CMC increase with increasing EO chain length. It occurs that the increasing EO chain length can increasingly penetrate and disrupt the closely packed surface monolayer, especially polyether-based trisiloxanes above 16 EO chain length. Moreover, the surface activities of poly(ethylene glycol) grafted polysiloxane were sensitive to the weight fraction of EO, the length of the EO chain, and size of the hydrophobic siloxane (trisiloxane, tetrasiloxane or polysiloxane) part. Like all non-ionics, siloxane surfactants are characterized by low critical micelle concentration values depending on both the polymerization and structure of hydrophilic and hydrophobic (methylsiloxane) groups [27].

Thanks to the specific and favorable physicochemical properties siloxane surfactants have been widely employed as stabilizers for polyurethane foams, antifoaming in fuels and heavy oils as well as polymer-based dispersions, detergents, paints and inks. They have also been proven to be efficient wetting agents in spreading aqueous formulations [28,29].

Although the range of practical application of these surfactants is so wide, the temperature effect on their stability as well as adsorption and micellization process in aqueous media has not been sufficiently established. In addition, the literature provides many discrepancies regarding the relationship between the structure and physicochemical properties of siloxane surfactants as well as their behavior in adsorption and micellization process at different temperatures. Some of the authors explain these discrepancies in terms of polydispersity of the surfactant.

Thus the purpose of our studies was to determine the adsorption and aggregation properties of freshly synthesized trisiloxane polyether surfactant: 3-[3-(hydroxy)(polyethoxy)propyl]- 1,1,1,3,5,5,5-heptamethyltrisiloxane) (TS-EO12). These properties were studied in wide range of TS-EO12 concentrations at different temperatures (293 K, 303 K and 313 K) by using surface tension, density as well as fluorescence intensities measurements. Additionally the morphology of the aggregates formed from the aqueous solution of studied surfactant with the concentration well above the CMC were studied by confocal microscopy. The synthesized products were characterized by ^1^H-NMR, ^13^C-NMR, ^29^Si-NMR, FT-IR and gel permeation chromatography (GPC) analysis. Thermal stability of TS-EO12 was characterized by using thermogravimetric analysis (TGA).

## 2. Results and Discussion

### 2.1. Synthesis Results

**Scheme 1:**
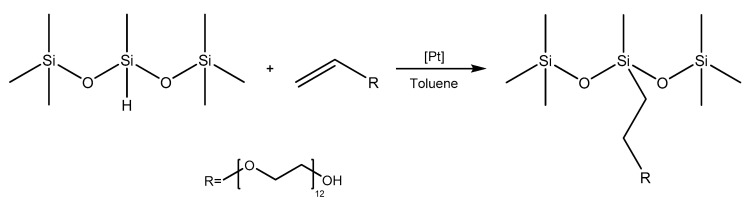
Hydrosilylation of allyl ether with 1,1,1,3,5,5,5-heptamethyltrisiloxane.

The siloxane polyether synthesis was performed using an allyl polyether containing 12 ethoxy groups with a terminal hydroxy group. Thus, two groups of contrasting surface properties, i.e., polyether groups showing hydrophilic properties and methyl groups in the siloxane showing hydrophobic properties, were linked in one compound. The reaction substrates, 1,1,1,3,5,5,5-heptamethyltrisiloxane and the olefin used are not sensitive to moisture, so the hydrosilylation reaction could be performed in an open system, which significantly facilitated the synthesis. All reactions were carried out in toluene as a solvent. The catalyst was the commercially available Karstedt one. The hydrosilylation reaction is shown in Scheme 1.

**Figure 1:**
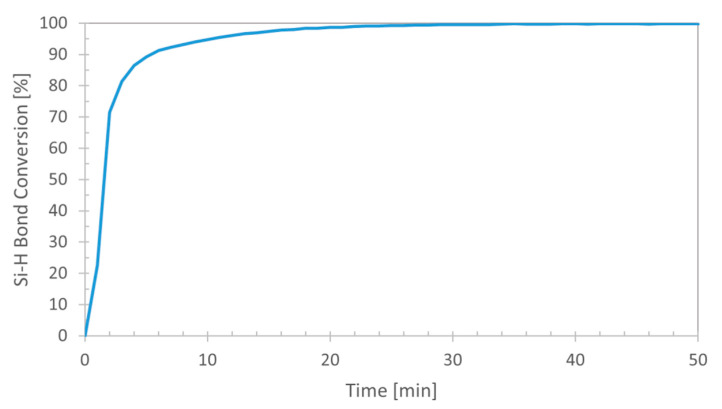
Surface plot illustrating the rate of changes in the area under the band at $\tilde{\upsilon }=904\;$ cm^−1^ for the catalytic system studied.

**Figure 2:**
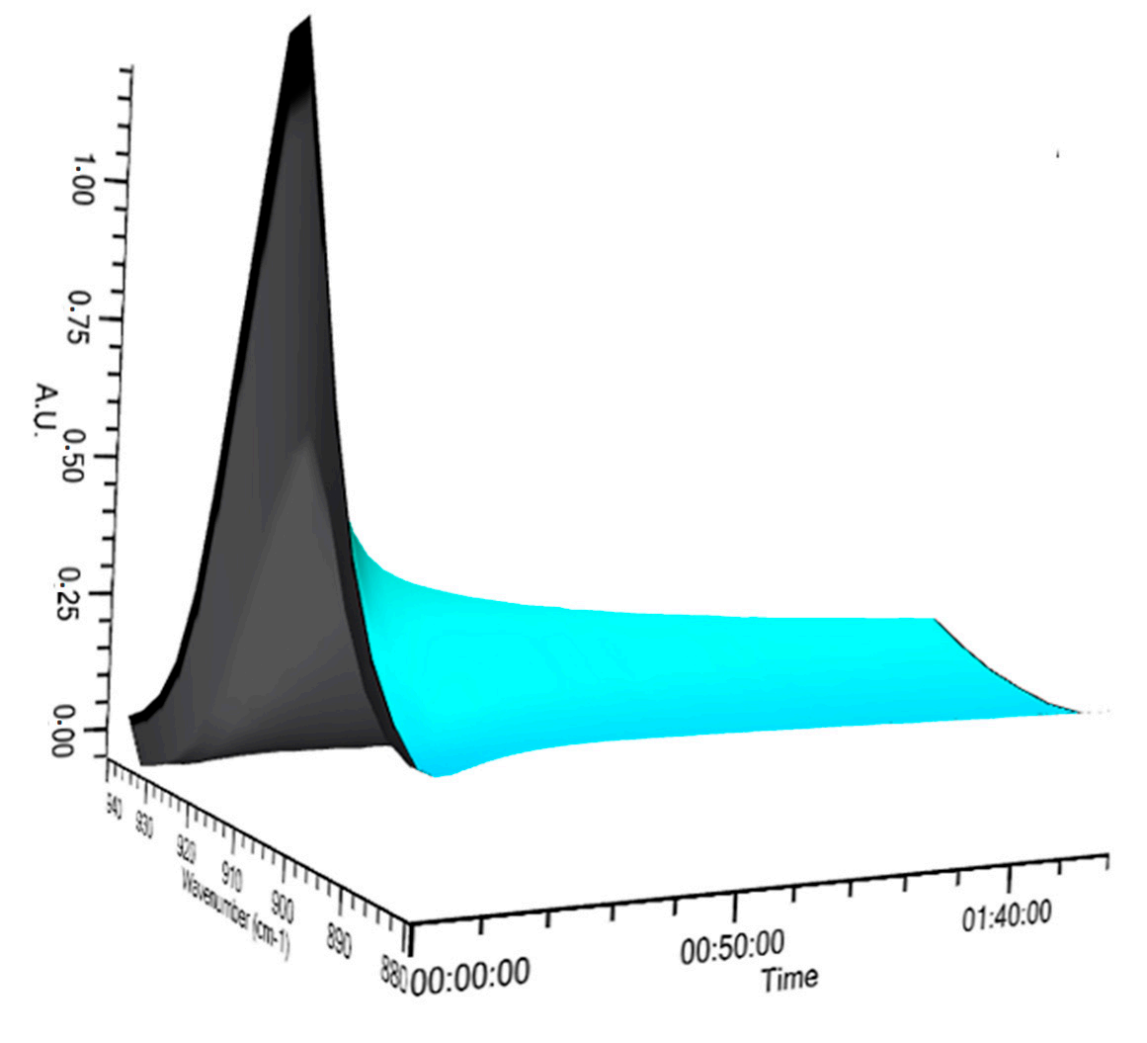
Analysis of real-time FTIR measurements showing changes in the intensity of the band at $\tilde{\upsilon }=904$ cm^−1^ with time during the hydrosilylation of 5-(allyloxy)-1,1,2,2,3,3,4,4-octafluoropentane in the presence of Karstedt’s catalyst.

**Figure 3:**
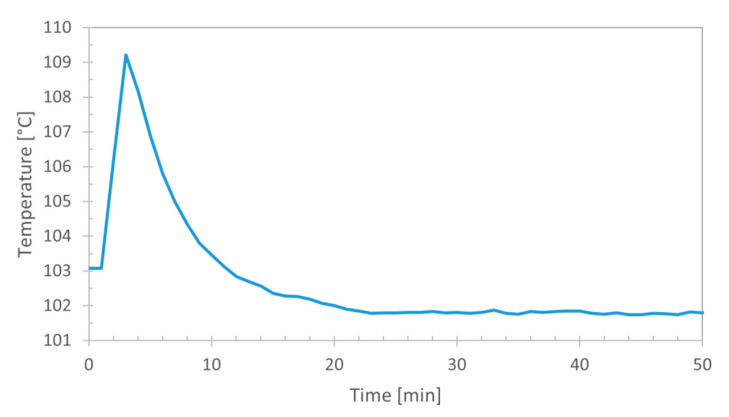
The course of hydrosilylation presented as the time dependence of temperature.

Hydrosilylation of allyl polyether with the terminal hydroxyl group with 1,1,1,3,5,5,5-heptamethyltrisiloxane was monitored using IR spectroscopy in real time. The reaction progress was quantified by checking the rate of changes in the area under the band with a maximum at a wavenumber ($\tilde{\upsilon }$) equal to 904 cm^−1^ assigned to the stretching vibrations of the Si–H bond, illustrated in Figure 1. In order to analyze the kinetics of the process in more detail, the trend of changes in the area of the observed band at 904 cm^−1^ in time is shown in Figure 2, while Figure 3 presents the trend of temperature changes in time. The parameters of the process were chosen so that the catalyst in a proper stoichiometric amount would be dosed after temperature stabilization. Analysis of the trends presented in Figure 2 and Figure 3, shows that the loss of Si–H bond conversion takes place directly after addition of a catalyst portion. At that time a jump in temperature, characteristic of exothermal hydrosilylation reaction takes place (Figure 3), leading to almost immediate conversion of the Si–H conversion reaching over 90% (Figure 2). Further increase in the Si–H conversion is induced by the heating of the reaction mixture as indicated by the correlated in time temperature change. Total conversion of Si–H bonds in the system studied was reached after about 55 min of the reaction measured from the moment of the catalyst addition. To sum up, the possibility of carrying the reaction out in an open system, high conversion reached in a relatively short time and high conversion of the process, reaching 97% indicate that the system is optimal and highly effective.

**Figure 4:**
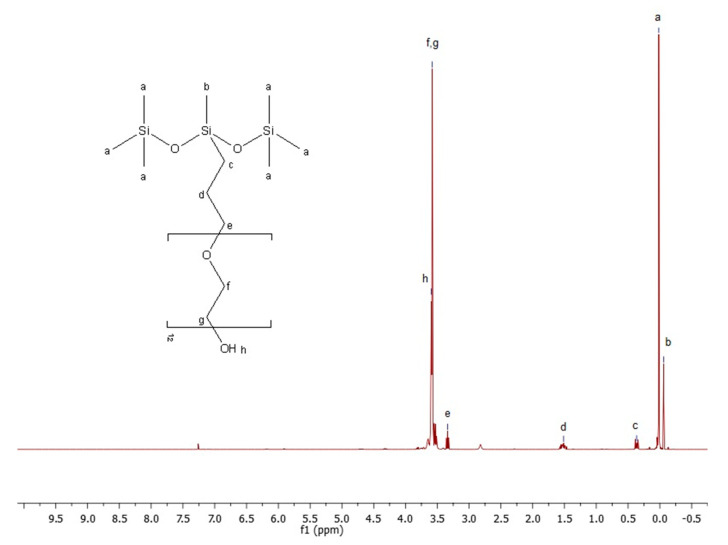
^1^H-NMR spectrum of the product.

**Figure 5:**
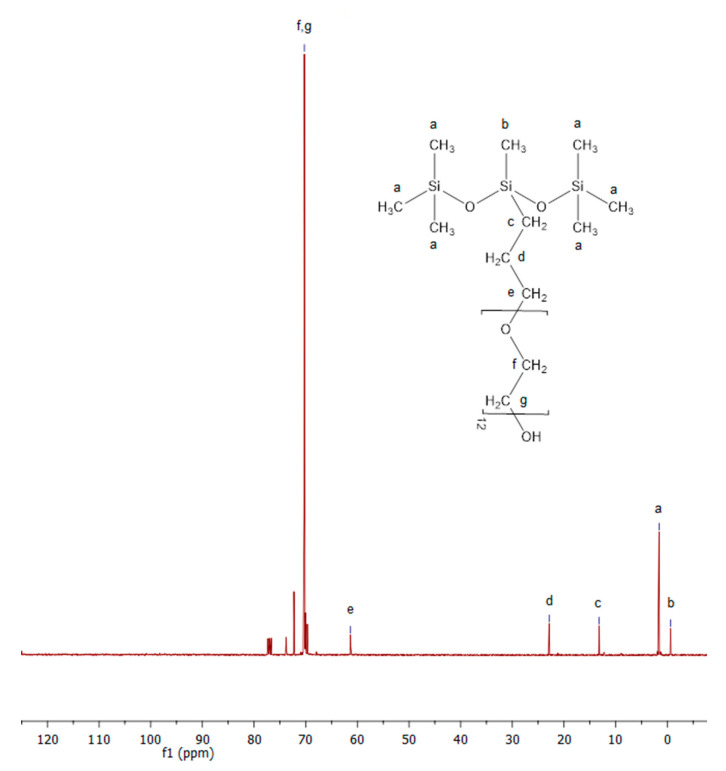
^13^C-NMR spectrum of the product.

**Figure 6:**
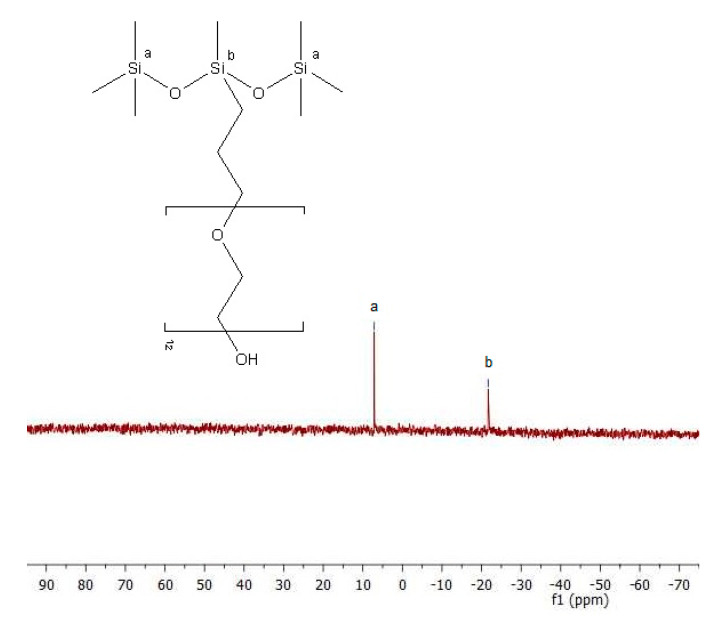
^29^Si-NMR spectrum of the product.

The isolated product was subjected to spectroscopic analysis (^1^H-NMR, ^13^C-NMR, ^29^Si-NMR) whose results are presented in Figure 4, Figure 5 and Figure 6. In the ^1^H-NMR spectrum of the product, 3-[3-(hydroxy)(polyethoxy)propyl]-1,1,1,3,5,5,5-heptamethyltrisiloxane (Figure 4), the characteristic signal at 5.15 ppm originating from the proton from Si–H bond disappeared. Also important is the lack of the characteristic bands assigned to the allyl groups, appearing at 5–6 ppm.

**Figure 7:**
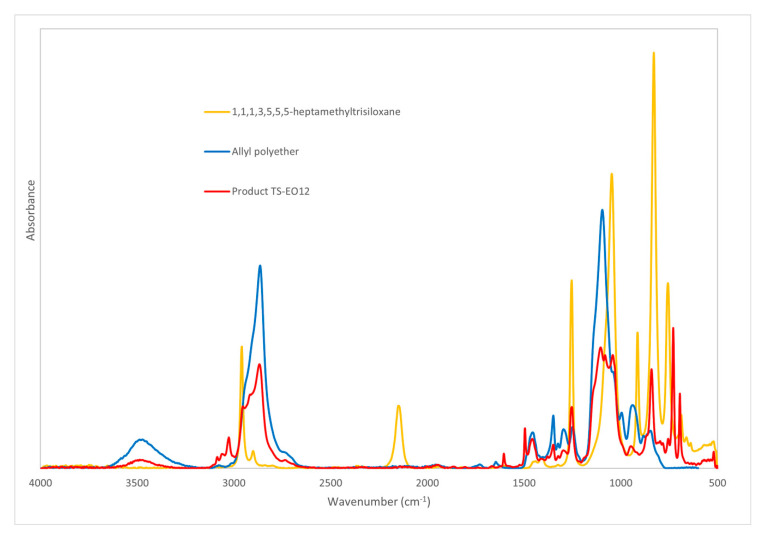
FT-IR spectrum of the product (red line) and the substrates 1,1,1,3,5,5,5-heptamethyltrisiloxane (yellow line) and allyl polyether (blue line).

The presence of the other bands confirms the complex structure of the product. In the ^29^Si-NMR spectrum (Figure 6) the presence of a signal near 7 ppm testifies to the presence of Si(Me)_3_O groups. The signal close to 21 ppm confirms that silicon occurs in different environment Si(CH_3_)CH_2_), which is characteristic for the compounds of this type. Formation of the product (Figure 7) of the desired structure is also confirmed by analysis of its FT-IR spectrum (red line) juxtaposed to the spectra of the substrates used (yellow and blue lines). The spectrum of the product (red line) shows a band at 3500 cm^−1^ characteristic for the stretching vibrations of hydroxyl groups (−OH).

The presence of this band in the spectrum, along with the disappearance of the bands at 2100 and 903 cm^−1^ assigned to the stretching vibrations of Si–H group present in the spectra of the substrate, testify to the formation of the product of hydrosilylation and not condensation of Si–H and −OH groups. The product structure is also confirmed by the presence of the overlapping symmetric and antisymmetric stretching vibrations, characteristic of C–H bonds of methyl and methylene groups in the range 2700–3000 cm^−1^ and the overlapping bands originating from the stretching vibrations of C–O–C bonds present in the polyether chains and asymmetric stretching vibrations of Si–O–Si bond in the range 1000–1200 cm^−1^.

**Figure 8:**
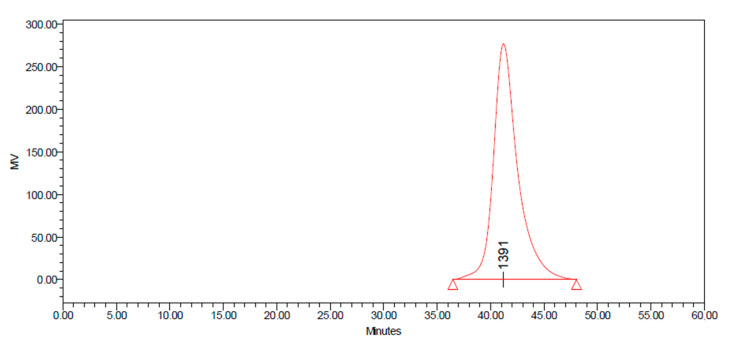
The GPC results of the product.

The molecular weight distributions of product was determined by GPC. The weight average molecular weight (MV) of functionalized siloxane was 1307 g/mol, with polymer dispersity index (PDI) of 1.353, indicating that the molecular weight distribution of product was relatively uniform (Figure 8).

**Figure 9:**
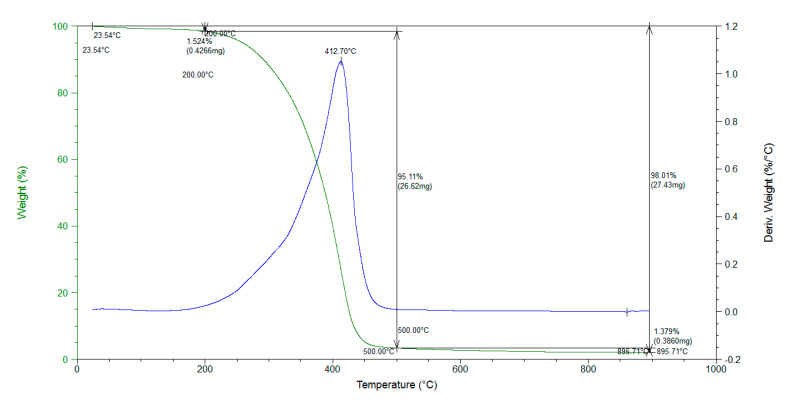
TGA and DTG curves of the product.

As shown in Figure 9, the thermogravimetry of the product was also investigated. In Figure 9 the TGA and DTG curves are compared. The polymer chains presented several decomposition stages. After an initial loss of solvents and absorbed water observed in sample, a decomposition step with complex shape was registered around 523.15 K and was assigned to cleavage of aliphatic fragments and ether bonds in the functional group and agree well with the molecular structure. Obviously, the siloxane backbone decomposes in higher temperature. The maximum weight loss appeared above 685.15 K.

There is only 1.37% of residue, which could be explained by the fact that siloxane backbone was decomposed into dimethylcyclosiloxane (DMC) at around 773.15 K, even under a nitrogen atmosphere, and pulled out along with the carrier gas. The decomposition mechanism seems to be affected by the chain structure and distribution. The decomposition temperature of a polymer is determined by the thermal stability of the molecules.

### 2.2. Equilibrium Surface Tension

From the practical point of view not only the knowledge of thermal stability of studied surfactant but also the temperature effect on the surface and aggregation properties are very desirable. They can be very helpful for understanding and explaining the large activity of TS-EO12 at the water-air interface under different conditions as well as low sensitivity of these surfactant properties with temperature.

In the case of siloxane surfactants the properties of the adsorbed monolayer at the water-air interface, which influences the changes in surface tension of their aqueous solutions, might depend on the structure and polymerization degree of the methylated siloxane hydrophobic moiety [27] as well as on the type and length of a polyether polar chain of surfactant.

**Figure 10:**
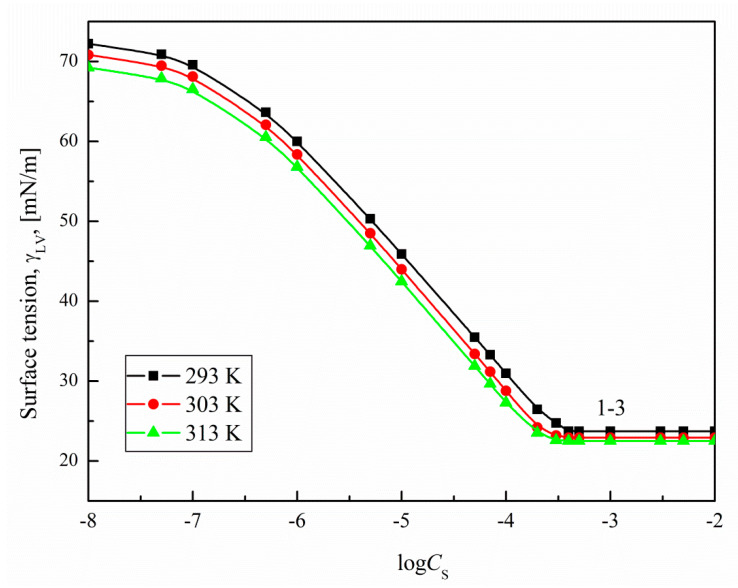
Measured values of surface tension ($\gamma_{LV}$) of aqueous solutions of TS-OE12 (curves 1–3) at 298 K, 303 K and 313 K vs. the logarithm of surfactant concentration (log*C_S_*).

Determination of the properties of the adsorbed monolayer of 3-[3-(hydroxy)(polyethoxy) propyl]-1,1,1,3,5,5,5-heptamethyltrisiloxane) (TS-EO12) (Scheme 1) as well as the thermodynamics of its adsorption at the water-air interface, requires the knowledge of the surface tension of aqueous solutions of the studied surfactant at different temperatures. The measured values of the equilibrium surface tension ($\gamma_{LV}$) of the studied surfactant aqueous solutions at 293 K, 303 K and 313 K are presented in Figure 10.

As follows from this figure, at all studied temperatures, the surface tension of the studied trisiloxane surfactant aqueous solutions decreases with increasing surfactant concentration (*C_S_*). It is similar as in the case of classical hydrocarbon surfactants as well as some others. In addition the surface tension of aqueous solutions of TS-EO12 depends quite strongly on temperature and increases with decreasing temperature. These results are in a good agreement with the data presented earlier for other siloxane surfactants [24,31] as well as many classical hydrocarbon surfactants and biosurfactants [32,33,34]. However, they are in contrast to those of some different siloxane surfactants [35] whose surface tension increases with increasing temperature.

For TS-EO12 aqueous solutions the minimal surface tension decreases with increasing temperature and changes from 23.7 mN/m at 293 K to 22.5 mN/m in 313 K. It is worth noting that the changes in the surface tension of TS-OE12 aqueous solutions with temperature are similar to those of pure POE (wt. 3400 gmol^−1^) aqueous solutions [35]. This fact suggests that the orientation of TS-EO12 molecules at the water-air interface can change with temperature. Probably the OE chain in the surfactant molecule is bent or crooked at the water-air interface. As mentioned above the temperature influences the orientation of the TS-EO12 molecules at the water-air interface, which is also connected with the area occupied by a surfactant molecule at the interface or the surfactant surface excess concentration.

### 2.3. Surface Excess Concentration of TS-EO12 at the Water-Air Interface at Different Temperatures

In order to calculate the amount of the adsorbed studied surfactants per unit area at the air-water interface (the Gibbs surface excess concentration of surfactant at the water-air interface (Γ*_S_*), the measured equilibrium surface tension ($\gamma_{LV}$) values (Figure 10) were treated in terms of the Gibbs adsorption equation [36]. This equation for dilute solutions (10^−2^ M/dm^3^ or less) of surfactants has the following form [36]:

(1) $$\Gamma_{S}=-\frac{C_{S}}{nRT}{\left(\frac{\partial \gamma_{LV}}{\partial C_{S}}\right)}_{T}=-\frac{1}{nRT}{\left(\frac{\partial \gamma_{LV}}{\partial lnC_{S}}\right)}_{T}=-\frac{1}{2.303nRT}{\left(\frac{\partial \gamma_{LV}}{\partial \log C_{S}}\right)}_{T}$$

where *R* is the gas constant, *T* is the absolute temperature, $\gamma_{LV}$ is the surfactant solution surface tension, *C_S_* is the concentration of surfactant and *n* is the constant which depends on the kind of surfactant (for studied nonionic trisiloxane surfactant it is equal to 1).

Γ*_S_* of the studied surfactants corresponding to the unsaturated adsorption monolayer at the water-air interface was determined from Equation (1) and using the second order polynomial equations describing the relationship between $\gamma_{LV}$ and *C_S_* of a given surfactant at a given temperature. The maximal Γ*_S_* values ($\Gamma_{S}^{\max}$) corresponding to the saturated adsorption monolayer of TS-OE12 at the water-air interface (Table 1) at a given temperature were determined from the linear dependence of $\gamma_{LV}$ vs. log*C_S_* and it is the parameter describing the effectiveness of a given surfactant adsorption at the water-air interface [36]:

(2) $$A_{S}^{\min}=\frac{1}{N_{A\;}\Gamma_{S}^{\max}}$$

where *N_A_* is Avogadro’s number.

**Figure 11:**
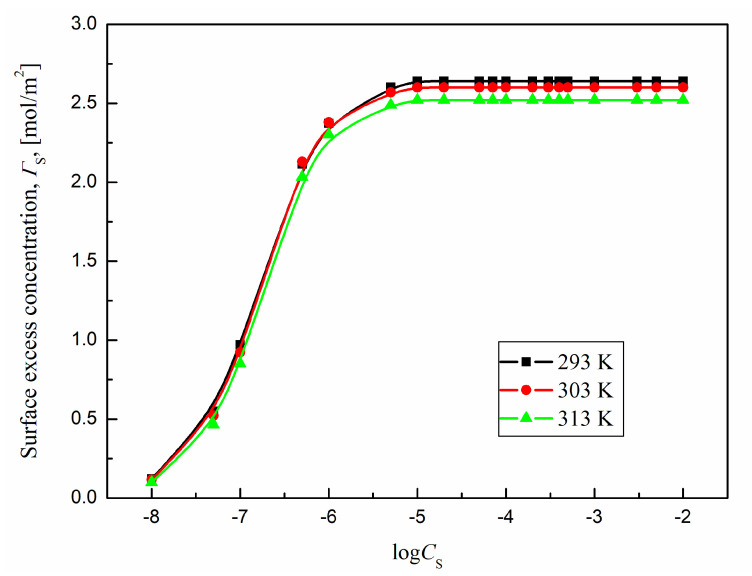
TS-OE12 Gibbs surface excess concentration (Γ*_S_*) at 298 K, 303 K and 313 K vs. the logarithm of surfactant concentration (log*C_S_*).

The changes in Γ*_S_* values of TS-OE12 at 293 K, 303 K and 313 K with the surfactant concentration in the bulk phase are presented in Figure 11. The $\Gamma_{S}^{\max}$ and $A_{S}^{\min}$ values calculated from Equations (1) and (2) for TS-OE12 at different temperatures are presented in Table 1 and they are close to those reported in literature [37]. It is difficult to compare the obtained $\Gamma_{S}^{\max}$ values at different temperatures with those reported in literature because of the lack of such data.

On the basis of our results it can be concluded that the minimal area per TS-OE12 molecule at the water-air interface rises with increasing temperature and according to Equation (2) it leads to a decrease in the maximal surface excess concentration of the studied surfactant at the interface with increasing temperature. However the changes in $\Gamma_{S}^{\max}$ and $A_{S}^{\min}$ with growing temperature are not significant (Table 1).

In addition, using the values of Γ*_S_* obtained from Equation (1) in a wide concentration range of TS-OE12 at different temperatures, the mole fraction of the area occupied by the molecules of the studied surfactant in the adsorption layer (*X_S_*) at a given temperature was determined from the relation [36]:

(3) $$\Gamma_{W}N_{A\;}A_{W}^{0}+\sum \Gamma_{S}N_{A\;}A_{S}^{0}=1$$

where Γ*_W_* and Γ*_S_* are the surface excess concentrations of water and surfactant at the water-air interface, $A_{W}^{0}$ and $A_{S}^{0}$ are the limiting areas of water or surfactant molecule at the water-air interface, respectively and:

(4) $$X_{S}=\frac{\Gamma_{S}}{\sum \Gamma_{S}+\Gamma_{W}}$$

For calculations of *X_S_* a proper $A_{W}^{0}$ value at a given temperature was used. *A_W_* values at different temperatures were calculated taking into account the increase of the distance between the water molecules in the surface region.

The value of $A_{S}^{0}$ (Table 1) can be determined from the Joos equation of state which for the aqueous solutions of surfactants can be written in the form [38]:

(5) $$exp\left(\frac{-\pi }{RT\Gamma_{W}^{\infty }}\right)+exp\left(\frac{-\pi }{RT\Gamma_{S}^{\infty }}\right)\frac{C_{S}}{a_{S}^{s}}=1$$

where $\Gamma_{W}^{\infty }$ and $\Gamma_{S}^{\infty }$ are the limiting Gibbs surface excess concentrations of water and surfactant at the water-air interface, respectively, *π* is the film pressure and $a_{S}^{s}$ is the activity of a given surfactant at the water-air interface.

The *X_S_* values against log*C_S_* for TS-OE12 obtained from Equation (4) at different temperatures are presented in Figure S1 (Supplementary Materials).

As follows from the obtained values, *X_S_* only slightly increases with temperature but its maximal value is close to 1 and suggest that the adsorption layer of TS-OE12 at the water-air interface is more densely packed than, for example, that of hydrocarbon surfactants [32,33,34].

On the other hand, as follows from Table 1, the $\Gamma_{S}^{\infty }$ values of the studied surfactant calculated from Equation (5) are somewhat higher than those of $\Gamma_{S}^{\max}$ in every case. Thus, on the basis of ΓSΓS∞ , it is possible to determine the extent of coverage at the water-air interface by the surfactant molecules and the obtained values should be equal to those calculated from Equation (4) [39]. The values of *X_S_* of particular surfactants obtained from the ratio ΓSΓS∞   are significantly higher than those calculated from Equation (4). As mentioned in earlier studies [39] the discrepancies can result from different values of the surface area occupied by the water and surfactant molecules because one molecule of surfactant can replace more than one molecule of water. The number of water molecules which can be replaced by one surfactant molecule at the water-air interface can be equal to the ratio of ΓS∞ΓW∞ .

Assuming that ΓS∞ΓW∞=1k [33,36,39], Equation (4) can be written as follows:

(6) $$X_{S}=\frac{1}{k}\frac{\Gamma_{S}}{\Gamma_{W}+\Gamma_{S}}$$

**Figure 12:**
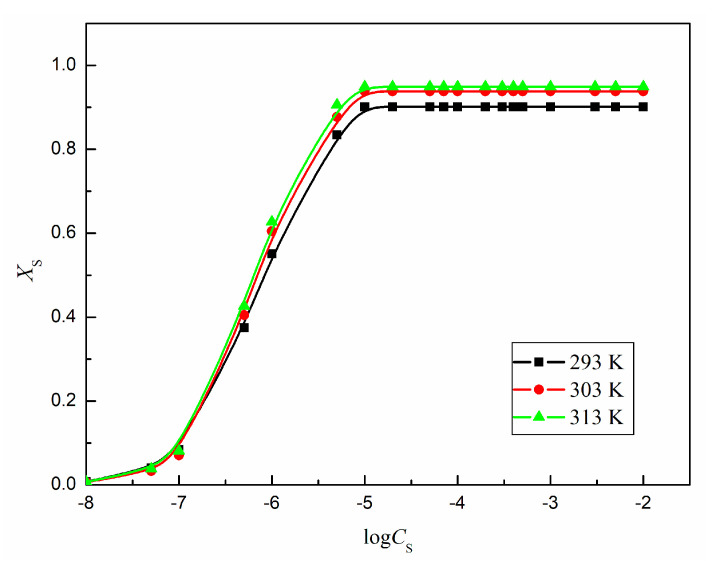
Mole fraction of the area occupied by TS-OE12 at the water-air interface (*X_S_*) vs. the logarithm of its concentration (log*C_S_*) at 293 K, 303 K and 313 K, determined from ΓSΓS∞ .

The values of *X_S_* for TS-OE12 calculated from Equation (6) are practically the same as those obtained from the ratio ΓSΓS∞  (Figure 12 and Figure S2). In addition, all isotherms of log*X_S_* for TS-OE12 in the studied temperature range are of the Langmuir type, which indicates that the adsorbed monolayer is formed at the water-air interface (Figure S3).

Analysis of the *X_S_* values of TS-OE12 and their changes with temperature (in the studied range) indicates that the dehydration process of polar heads probably proceeds to a smaller extent. Further consideration of the TS-OE12 adsorption at the water-air interface changes with temperature requires the knowledge of changes in the standard Gibbs free energy, enthalpy and entropy of adsorption.

It is also interesting from the practical point of view, whether it is possible to describe the changes in the surface tension of aqueous solutions of the studied surfactant as a function of its concentration on the basis of the solution components’ properties.

For this purpose the Szyszkowski equation [36,40] was used:

(7) $$\gamma_{W}-\gamma_{LV}=RT\Gamma_{S}^{\max}ln\left(1+\frac{C_{S}}{b}\right)$$

where $\Gamma_{S}^{\max}$ is the maximal surface excess of the studied surfactant concentration in the surface layer,$\;\gamma_{W}$ is the water surface tension and *b* is a constant.

To solve Equation (7) the maximal and limiting Gibbs surface excess concentrations of TS-OE12 (Table 1) were used.

Similarly as for the other quite different surfactants [41,42], the use of the limiting area of the surfactant at the water-air interface instead of the minimal one, gives better agreement of $\gamma_{LV}$ values measured and those calculated from Equation (7) (Figure S4).

### 2.4. Critical Micelle Concentration (CMC) of TS-OE12

**Figure 13:**
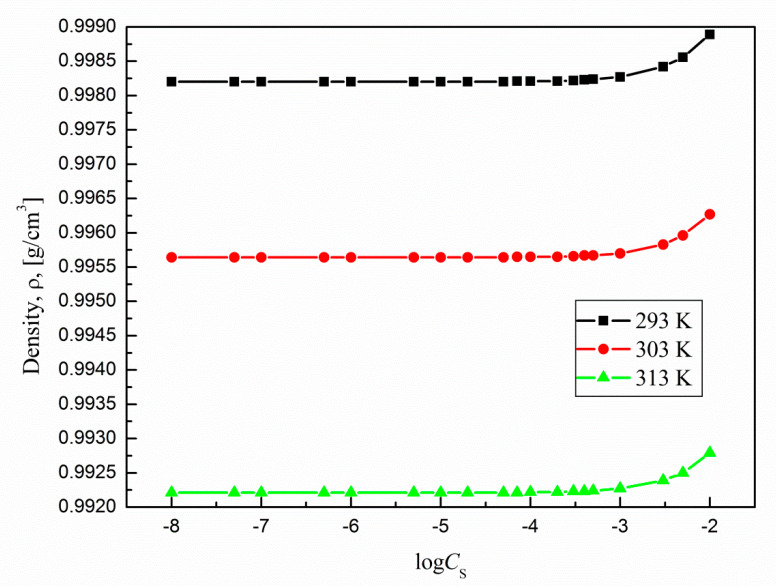
Density (*ρ*) of aqueous solutions of TS-OE12 at 298 K, 303 K and 313 K vs. the logarithm of surfactant concentration (log*C_S_*).

**Figure 14:**
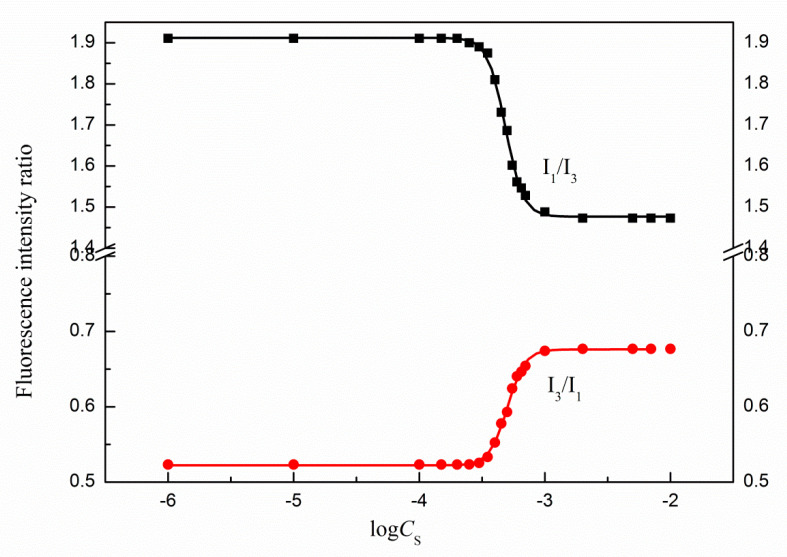
The ratios of *I*_1_/*I*_3_ and *I*_3_/*I*_1_ for pyrene vs. the logarithm of TS-OE12 concentration (log*C_S_*) at 298 K.

Based on the literature data it is difficult to find systematic informations about the mechanism of temperature effect on siloxane surfactants aggregation properties. Thus, at the beginning, the critical micelle concentration (CMC) of TS-OE12 in aqueous solutions were determined at different temperatures (293 K, 303 K and 313 K) by using different methods. Of all the methods that can be used for estimation of CMC the surface tension method is the most popular. In this method the CMC value can be determined from plots of surface tension versus log of surfactant concentration. In our studies the CMC of TS-OE12 at a given temperature was also determined on the basis of the density (*ρ*) (Figure 13) as well as pyrene (Py) (Figure 14) fluorescence intensity values changes as a function of the surfactant concentration in the solution. In the case of the Py fluorescence intensity measurements the CMC values of studied surfactants were determined from the changes in the first and third vibronic bands ratio (*I*_1_/*I*_3_) on the pyrene fluorescence spectra with the surfactant concentration in the solution.

The CMC values obtained in such a way for the studied surfactant are presented in Table 1. According to Table 1, TS-OE12 is not characterized by a single CMC value but can assume values from a certain range and depends on the method of its determination. The obtained CMC values for TS-OE12 are consistent with literature data [24,27]. However, it is difficult to compare all the obtained values of CMC in different temperatures because of the lack of such data.

The Table 1 data also imply that CMC of TS-OE12 determined from the surface tension measurements decreases slightly with increasing temperature. This decrease can be connected with the dehydration process and the loss of hydration water. On the other hand the rise of temperature increases the flexibility of Si–O–Si linkage and extends the minimal area of surfactant at the water-air interface. This conclusion was also deduced from the spectroscopic measurement results as well as thermodynamic considerations.

The CMC values of TS-OE12 were also determined from the changes of the first and third vibronic bands ratio on the pyrene fluorescence spectra with the surfactant concentration in the solution (Figure 14, Figures S5 and S6). Pyrene is a strongly hydrophobic fluorescence probe and its solubility in water is in the range of 2–3 μM. Thus, in the presence of micelles, pyrene is preferentially solubilized in the interior hydrophobic regions of these micelles. This is the basis of successful use of pyrene as a fluorescence probe in the study of micellization process [43]. For TS-OE12, like for the other types of surfactants, the changes in the ratio *I*_1_/*I*_3_ as a function of surfactant concentration can be described by the decreasing Boltzman sigmoid (Figure 14, Figures S5 and S6). As follows from the fluorescence measurements also for TS-OE12 its CMC decreases with temperature (Table 1).

For pyrene, the ratio *I*_1_/*I*_3_ takes values of 1.04, 1.33 and 1.84 for toluene, methanol and water, respectively. The values of *I*_1_/*I*_3_ for TS-OE12 presented in Figure 14 and Figures S5 and S6 are in the range from 1.9 (no micelles) to about 1.5 (micellar solution) and decrease with increasing temperature. Above CMC it remains nearly constant and independent of the surfactant concentration, which confirms that the probe is located close to the micellar surface and the temperature increase induces changes in the *I*_1_/*I*_3_ values in the same direction as the CMC changes. In addition, it confirms high water penetration into the so-called palisade layer of the micelle and lose packing of the SF-OE12 micelles. As results from the changes in the ratio *I*_1_/*I*_3_ with the logarithm of the surfactant concentration at a given temperature, pyrene is a sensitive probe for determination of the TS-OE12 CMC. In addition, as follows from Figure 12, Figures S5 and S6, and from the changes in the third and first vibronic bands ratio, with increasing temperature the hydrophobicity of the interior of the studied micelles increases. However, this effect is not significant in the studied temperature range.

**Figure 15:**
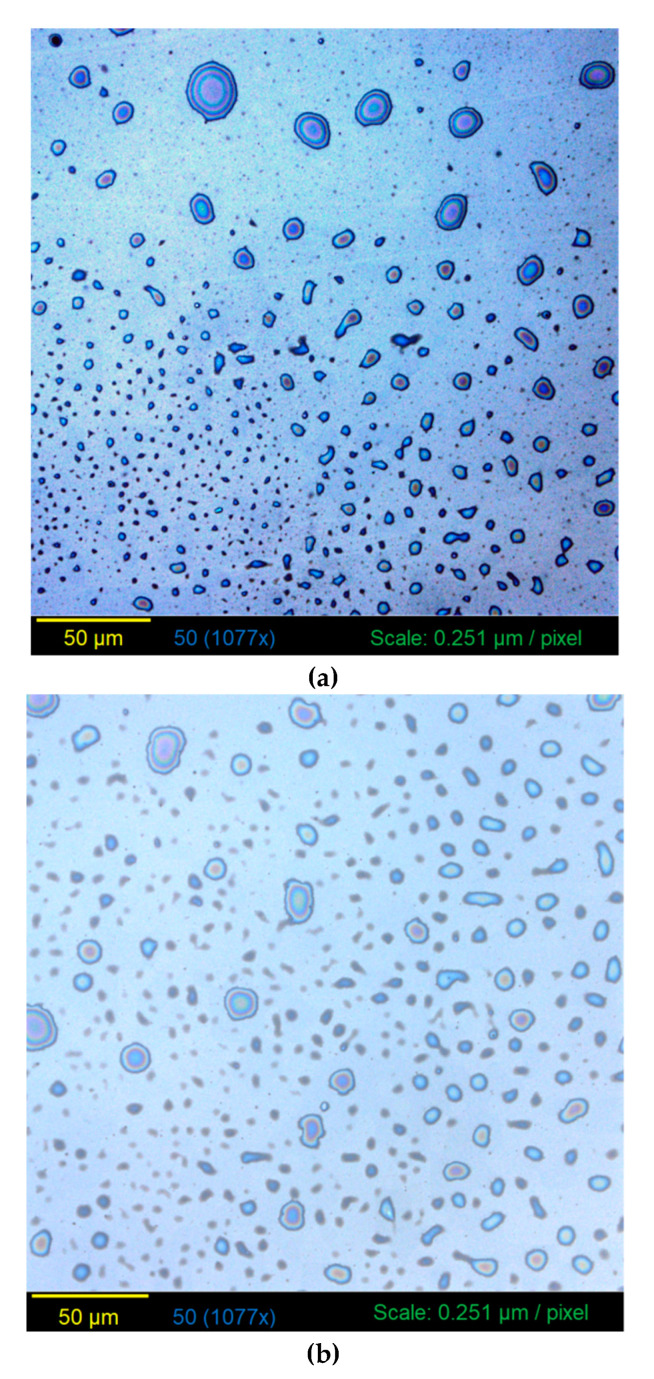
(**a**) Confocal microscopy images of glass plates modified with TS-OE12 aqueous solutions by sonication (GPM1) method. (**b**) Confocal microscopy images of glass plates modified with TS-OE12 aqueous solutions by immersing (GPM2) method.

Additionally the morphology of TS-OE12 aggregates in the aqueous solution at the surfactant concentration well above its CMC (and at 293 K) was determined by using the confocal microscopy (Figure 15a,b). From this analysis it results that aggregates of the surfactant could be considered as more or less spherical or ellipsoidal ones. It occurred that similar shape of aggregates were obtained by using both methods of glass surface modification that is sonication and immersing method. These findings demonstrate the need for further analysis of TS-OE12 aggregates structure.

Analysis of the changes in CMC and hydrophobicity of the interior of TS-OE12 micelles should be supplemented with thermodynamic considerations taking into account the standard Gibbs free energy, enthalpy and entropy of micellization as well as temperature changes in molar volume.

### 2.5. Apparent and Partial Molar Volume

The micellization process should be also reflected by changes in the surfactant aqueous solutions density (Figure 13). As follows from this figure, the slopes of the plots of density vs concentration of surfactants before and after CMC are different, which allows determination of CMC values for a studied surfactant (Table 1). However, the influence of surfactant concentration on the structure of the solution can be more visible from changes in the apparent ($\phi_{V}$) and partial (${\bar{V}}_{M}$) molar volumes with the surfactant concentration in the micellization process.

The apparent molar volume can be determined from the following equation [44,45]:

(8) $$\phi_{V}=\frac{M_{S}}{\rho_{0}}+\frac{1000\left(\rho_{0}-\rho \right)}{C}$$

where *M*_S_ is the molecular weight of the surface active agent, *C* is the concentration of the surface active agent in mol/cm^3^ and *ρ*_0_ is the density of the solvent.

The partial molar volume ${\bar{V}}_{M}\;$ can be calculated from the equation [45]:

(9) $${\bar{V}}_{M}=\frac{M_{S}}{\rho }\left[1-\frac{\left(100-C_{p}\right)}{\rho }\frac{d\rho }{dC_{p}}\right]$$

It appeared that the *ρ* data measured for TS-OE12 at different temperatures (Figure 13) fit a polynomial of *C_p_* (the mass percentage concentration) given by:

(10) $$\rho =a+bC_{p}+dC_{p}^{2}$$

where *a*, *b* and *d* are constants.

**Figure 16:**
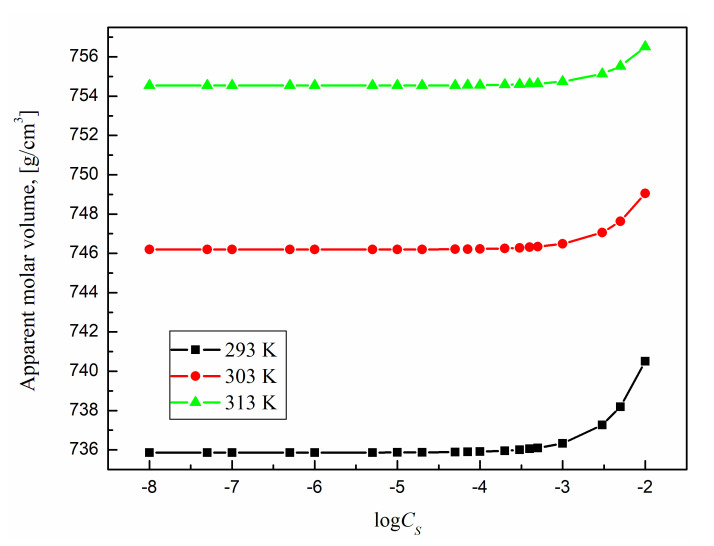
The apparent molar volume ($\phi_{V}$) of TS-OE12 vs. the logarithm of its concentration (log*C_S_*) at 293 K, 303 K and 313 K.

**Figure 17:**
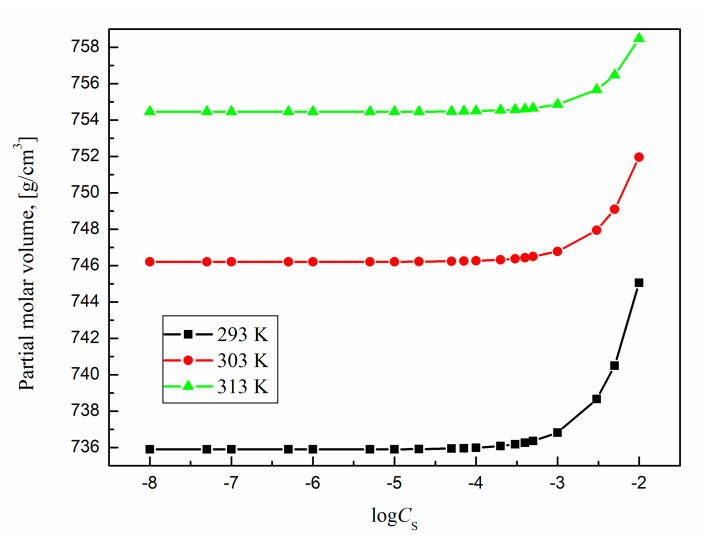
The partial molar volume (${\bar{V}}_{M}$) of TS-OE12 vs. the logarithm of its concentration (log*C_S_*) at 293 K, 303 K and 313 K.

On the basis of the above- mentioned relations between *ρ* and *C_p_*, the apparent ($\phi_{V}$) and partial (${\bar{V}}_{M}$) molar volumes as well as their changes with the surfactant concentration and temperature were determined (Figure 16 and Figure 17).

From these figures it results that the partial and apparent molar volume values for TS-OE12 increase with surfactant concentration and there is no characteristic point corresponding to CMC. The growth of density with TS-OE12 concentration (Figure 13) indicates also that the density of the micellar solution is higher than that of the surfactant in the monomeric form and that the size of micelles increases as a function of TS-OE12 concentration. It is possible to calculate the the size of aggregates as well as a mean aggregation number based on the length of the hydrophobic part of surfactant and its volume as well as on the basis of a diameter of the surfactant aggregate and molar volume of one molecule of surfactant determined from the density measurements. From Figure 15a,b it is difficult to state one unequivocal diameter value of the studied surfactant micelles. Thus additional measurements should be done. In addition the siloxane chains in the core part are expected to be in a coiled form because of the high flexibility of the Si–O–Si bonds.

According to Figure 16 and Figure 17, the values of ${\bar{V}}_{M}$ similarly as those of $\phi_{V}$ insignificantly increase with increasing temperature. This small increase probably results from the increase in the average distance between the surfactant and water molecules for the surfactant concentrations lower than CMC and between surfactant molecules in the micelles for the surfactant concentrations higher than CMC.

### 2.6. Thermodynamic Parameters of Adsorption and Micellization 

Determination of standard thermodynamic functions (Gibbs free energy, enthalpy and entropy) of the studied surfactants adsorption at the water-air interface gives us more information about the mechanism of the process. Similarly, the standard enthalpy of micellization ($\Delta H_{mic}^{0}$), Gibbs free energy of micellization ($\Delta G_{mic}^{0}$) and entropy of micellization ($\Delta S_{mic}^{0}$) are important in understanding micelle formation in aqueous solution.

For these reasons, on the basis of the dependence of Γ*_S_* on *C_S_* for TS-OE12 at different temperatures, the standard Gibbs free energy ($\Delta G_{ads}^{0}$) and next enthalpy ($\Delta H_{ads}^{0}$) and entropy ($\Delta S_{ads}^{0}$) of their adsorption at the water-air interface at a given temperature were determined. There are many approaches which can be used to determine $\Delta G_{ads}^{0}$ [36,40]. Among others, for dilute aqueous solutions whose concentration corresponds to the unsaturated monolayer at the water-air interface, the Langmuir equation can be used [36,40]. If mutual interactions between the adsorbed molecules are taken into account, the Langmuir equation is as follows [36]:

(11) $$\frac{A_{S}^{0}}{A_{S}-A_{S}^{0}}exp\frac{A_{S}^{0}}{A_{S}-A_{S}^{0}}=\frac{C}{\omega }exp\left(\frac{\Delta G_{ads}^{0}}{RT}\right)$$

where *A_S_* is the area occupied by the surfactant molecule at the interface, *ω* is the number of water moles in 1 dm^3^ (at a given temperature). To calculate $\Delta G_{ads}^{0}$ from Equation (11), the values of $A_{S}^{0}$ for a given surfactant must be known. These values were determined earlier from Equation (5) (Table 2). The values of *A_S_* at the water-air interface for studied surfactants at a given temperature were determined from the appropriate values of Γ*_S_* (Figure 11). The obtained $\Delta G_{ads}^{0}$ values of TS-OE12 are presented in Table 2.

As follows from this table, the $\Delta G_{ads}^{0}$ values calculated from Equation (11) are constant only in the range of surfactants concentrations corresponding to the unsaturated monolayer at the water-air interface and the $\Delta G_{ads}^{0}$ values decrease with increasing temperature. According to the thermodynamic rule $\Delta G_{ads}^{0}$ fulfils the relationship [36,40]:

(12) $$\Delta G_{ads}^{0}=\Delta H_{ads}^{0}-T\Delta S_{ads}^{0}$$

$\Delta S_{ads}^{0}$ can be calculated from the relation:

(13) $$\frac{d\Delta G_{ads}^{0}}{dT}=-\Delta S_{ads}^{0}$$

if $\Delta H_{ads}^{0}$ is constant over the investigated temperature range. To calculate the standard enthalpy of adsorption of the studied surfactant, the standard entropy of adsorption determined from the $\Delta G_{ads}^{0}$ (from Equation (11)) linear changes with temperature in the studied temperature range was used. The obtained values of $\Delta H_{ads}^{0}$ and $\Delta S_{ads}^{0}$ for TS-OE12 are presented in Table 2. The obtained values of standard Gibbs free energy ($\Delta G_{ads}^{0}$), enthalpy ($\Delta H_{ads}^{0}$) and entropy ($\Delta S_{ads}^{0}$) of TS-OE12 adsorption at the water-air interface indicate the positive values of $\Delta S_{ads}^{0}$. As the $\Delta H_{mic}^{0}$ values are influenced by bonds formation, thus positive values of standard enthalpy and entropy of TS-OE12 adsorption suggest that bond breaking predominates in the adsorption process. In addition, the $\Delta H_{ads}^{0}$ values suggest that in the studied temperature range, dehydration of the TS-OE12 molecules takes place (Table 2).

There are also different approaches to determination of $\Delta G_{mic}^{0}$. The phase separation model is the simplest one and treats micelles as a simple phase. In this model, micelle formation is treated as a phase separation phenomenon. In the mass action model, micelles and single surfactant molecules or ions are assumed to be in the association-dissociation equilibrium [40].

According to the micellization process, for nonionic surfactants such as TS-OE12, $\Delta G_{mic}^{0}$ assumes the following form [36]:

(14) $$\Delta G_{mic}^{0}=RTln\frac{CMC}{\omega }$$

According to the thermodynamic rule, in the exothermic and isobaric conditions, $\Delta G_{ads}^{0}$ fulfills the relationship [36,40]:

(15) $$\Delta G_{mic}^{0}=\Delta H_{mic}^{0}-T\Delta S_{mic}^{0}$$

Knowing the values of the standard Gibbs free energy of micellization at different temperatures it is possible to determine the standard enthalpy and entropy of micellization.

$\Delta S_{mic}^{0}$ can be calculated from the relation:

(16) $$\frac{d\Delta G_{mic}^{0}}{dT}=-\Delta S_{mic}^{0}$$

if $\Delta H_{mic}^{0}$ is constant over the investigated temperature range.

Alternatively, when $\Delta S_{mic}^{0}$ is constant over the temperature range investigated, then it is possible to calculate $\Delta H_{mic}^{0}$ from the following equation:

(17) $$T^{2}\frac{d\left(\frac{\Delta G_{mic}^{0}}{T}\right)}{dT}=-\Delta H_{mic}^{0}$$

As mentioned above, there are some differences in CMC for the same surfactant determined from the surface tension, density and spectrofluorimetric measurements (Table 2). In the calculations of $\Delta G_{mic}^{0}$ of TS-OE12 only the CMC values obtained from the surface tension measurements were taken into account. The obtained $\Delta G_{mic}^{0}$ values are presented in Table 2.

It occurs that the Gibbs free energy of micellization becomes more negative as the temperature increases, indicating that the formation of micelle becomes more spontaneous at higher temperatures. Next, taking into account the relation between $\Delta G_{mic}^{0}$ and *T*, it was possible to determine $\Delta S_{mic}^{0}$ of micellization of TS-OE12 assuming that $\Delta H_{mic}^{0}$ of micellization in the studied range of temperatures is constant. It appeared that there is a linear dependence between $\Delta G_{mic}^{0}$ and temperature. The slope of this dependence is equal to $–\Delta S_{mic}^{0}$. Knowing the values of $\Delta S_{mic}^{0}$, $\Delta H_{mic}^{0}$ were found from Equation (17). The obtained values of $\Delta S_{mic}^{0}$ and $\Delta H_{mic}^{0}$ are presented in Table 2. According to this table, the standard enthalpy and entropy of TS-OE12 micellization take positive values and those of $\Delta S_{mic}^{0}$ are higher than zero. The values of $\Delta H_{mic}^{0}$ indicate that the micellization process is endothermic. This parameter shows a very strong dependence on temperature, indicating changes in the environment surrounding the hydrocarbon chain of the surfactant molecule with temperature changes. It is also known that $\Delta H_{mic}^{0}$ indicates whether during the micellization process more bonds are formed than broken.

## 3. Materials and Methods

### 3.1. Materials

All commercially available chemicals were used as received without any further purification. 1,1,1,3,5,5,5-heptamethyltrisiloxane was purchased from Sigma-Aldrich (St. Louis, MO, USA). Allyl polyether was purchased from ICSO Chemical Production (Kędzierzyn-Koźle, Poland). The hydrosilylation catalyst was a commercially available Karstedt catalyst purchased from Sigma-Aldrich.

Doubly distilled and deionized water (Destamat Bi18E, Heraeus, Hanau, Germany) was used for 3-[3-(hydroxy)(polyethoxy)propyl]-1,1,1,3,5,5,5-heptamethyltrisiloxane) (TS-EO12) solutions preparation. Pyrene (Py, purity > 99%) was purchased from Sigma-Aldrich and used without further purification. Ethanol (EtOH) for the pyrene stock solutions preparation was purchased from Avantor Performance Materials Poland S.A. (Gliwice, Poland). The ethanol stock solution of pyrene as well as aqueous stock solutions of TS-EO12 of the appropriate concentrations were prepared by weighing using an analytical balance (model XA105, Mettler-Toledo, Columbus, OH, USA). All the aqueous solutions at a given surfactant concentration lower than that at the stock solution were prepared by dilution of the appropriate stock solution. The pyrene concentration in the stock ethanol solution and in the surfactant solution was equal to 1 × 10^−2^ mol/dm3 and 2 × 10^−6^ mol/dm^3^, respectively.

### 3.2. Methods

Magnetic Nuclear Resonance spectra: ^1^H-NMR, ^13^C-NMR and ^29^Si-NMR were taken on an Ascent 400 instrument (Bruker, Billica, MA, USA) at room temperature, with CDCl_3_ as a solvent. FT-IR spectra were recorded on a Bruker Tensor 27 spectrophotometer equipped with a SPECAC Golden Gate diamond ATR attachment. The spectra were collected in the range 500–4000 cm^−1^, with the resolution of 2 cm^−1^, recording always 16 scans of the background and the sample. The hydrosilylation process was monitored with IR spectroscopy in real time. The measurements were made using a ReactIR 15 spectrometer (Mettler Toledo), equipped with a 9-unit reflection probe with an AgX DiComp diamond window of 9.5 mm (Mettler Toledo) and an MCT detector cooled with nitrogen. The spectra were recorded at a resolution of 4 cm^−1^, accumulating 50 scans of each spectrum, at 15 s intervals. The reaction progress was quantified by observation of the rate of changes in the area of the band with a maximum at 904 cm^−1^, assigned to the stretching vibrations of Si–H.

The gel permeation chromatography (GPC) measurements were made using a 2414 RI detector (Waters, Milford, MA, USA) and three 7.8 × 300 mm columns connected in series (HR1, HR2 and HR4). THF with a flow of 0.6 mL/ min was used as the mobile phase, column temperature was 308.15 K (35 °C), detector temperature was 313.15 K (40 °C). All molecular weights and mass distribution coefficients (PDIs) were calculated from a calibration curve for polystyrene standards (Shodex, Tokyo, Japan) in the mass range from 1.31 × 103 to 3.64 × 106 Da. Thermogravimetric analysis (TGA) was performed on a Q50 apparatus (TA Instruments, New Castle, PA, USA), under nitrogen flow (60 mL/min) from room temperature to 1173.15 K (900 °C), at a heating rate of 283.15 K/min (10 °C/min).

The equilibrium surface tension of water and freshly prepared aqueous solutions of the studied surfactants was measured at 293 K, 303 K and 313 K by a KC100 tensiometer (Krüss, Hamburg, Germany) according to the platinum ring tensiometer (or du Nouy’s) method. The solution to be measured was transferred to the measurement vessel, taking care to avoid foaming. In addition, before the measurement the measurement vessel was washed with hot chromo-sulfuric acid and subsequently with syrupy phosphoric acid (83 to 98% by weight of H_3_PO_4_), thoroughly rinsed in tap water and finally washed with double distilled water. The measured surface tension values were corrected according to the procedure of Harkins and Jordan. They have empirically determined correction factors which are dependent on ring dimensions, the density of the liquid and its surface tension. The ring was cleaned with acetone and next with distilled water and heated to red color with a Bunsen burner before each measurement. In all cases more than 10 successive measurements were performed. The standard deviation depending on the surfactant concentration range was from ± 0.1 to ± 0.2 mN/m (depending on the surfactant concentration range). The temperature was controlled by a jacketed vessel joined to the thermostatic water bath with the accuracy ± 0.01 K.

The density of aqueous solutions of surfactants studied was measured with a U-tube densitometer (DMA 5000, Anton Paar, Graz, Austria) at constant temperatures equal to 293, 303 and 313 K. The accuracy of temperature and density measurements were ±0.01 K and ± 0.005 kg/m^3^, respectively. The precision of the density and temperature measurements declared by the manufacturer is ±0.001 kg/m^3^ and ±0.001 K. The densitometer was calibrated regularly with distilled and deionized water.

The fluorescence intensities of the probe (pyrene) in aqueous solutions of studied surfactant were measured at 293, 303 and 313 K on a F-2700 fluorescence spectrophotometer (Hitachi, Tokyo, Japan) using 10 mm quartz cell. A 300 nm/min scan speed was used.

The emission spectra of pyrene were monitored at the excitation wavelength at 335 nm and the excitation and emission slits were set at 2.5 nm. The intensities *I*_1_ and *I*_3_ were read at the wavelengths corresponding to the first and third vibronic bands close to 372 and 384 nm, respectively.

Morphology of TS-EO12 aggregates was characterized by a Quanta FEG 250 scanning electron microscope (FEI, Morristown, NJ, country) under high vacuum conditions using an accelerating voltage of 1.2 kV. Optical microscopy imaging was realized by a LEXT OLS4100 laser scanning confocal microscope (Olympus, Tokyo, Japan). In a both cases (SEM, optical microscopy) measurements were performed on a glass plate. For this purpose glass plates were modified in two different ways: by sonication and immersing methods. In the first way the surfactant solution (2 × 10^−2^ mol/dm^3^) was applied directly on a cleansed substrate by means of ultrasounds. The plates were placed in a beaker filled with a modifying compound solution and subjected to the action of ultrasound for 15 min. Glass plates were dried in a desiccator at room temperature for 24 h. In the second method glass plates were immersed in surfactant solution for 30 min. After this time, excess solution was removed and plates were dried in a desiccator at room temperature for 24 h.

### 3.3. Synthesis of 3-[3-(Hydroxy)(polyethoxyy)propyl]-1,1,1,3,5,5,5-Heptamethyltrisiloxane

The siloxane-containing polyether group was synthesized in the hydrosilylation reaction of 1,1,1,3,5,5,5-heptamethyltrisiloxane and allyl polyether containing twelve ethoxy groups with the terminal hydroxy group. The process was carried out in the presence of Karstedt catalyst and using toluene as a solvent. 1,1,1,3,5,5,5-heptamethyltrisiloxane was placed in a three-necked round-bottom flask equipped with a reflux, thermometer and magnetic stirrer and then dissolved in toluene. The mixture was heated to the process temperature of 383.15 K (110 °C), then the olefin was added in a stoichiometric amount. A portion of Karstedt catalyst corresponding to 1 × 10^−4^ mol Pt/ 1 mol of Si–H bonds, was added. The reaction mixture was maintained at 383.15 K (110 °C) until the substitution of the olefin. The course of the reaction was monitored by IR spectroscopy, by observation of disappearance of the band at 904 cm^−1^, assigned to the Si–H bond in the substrate. After completion of the process, the post-reaction mixture was cooled and the products were isolated by distilling off the solvent and excessive olefin under reduced pressure. The pure product was obtained in high yields of 97%. The products were subjected to spectroscopic analysis to verify obtainment of the assumed structure.

### 3.4. Product Characterization

^1^H-NMR (CDCl_3_, TMS) δ (ppm): −0.06 (–Si(CH_3_)_3_); 0.02 (–SiCH_3_); 0.36 (–SiCH_2_–); 1.51 (–CH_2_CH_2_CH_2_–); 3.34 (–CH_2_O–); 3.59 (–OCH_2_CH_2_–); 3.61 (–OH).

^13^C-NMR (CDCl_3,_ TMS) δ (ppm): −0.65 (–SiCH_3_); 1.59 (–Si(CH_3_)_3_); 13.19 (–SiCH_2_–); 22.81 (–CH_2_CH_2_CH_2_–); 61.32 (–CH_2_O–); 70.27 (–OCH_2_CH_2_–).

^29^Si-NMR (CDCl_3,_ TMS) δ (ppm): −21.93 (–Si(CH_3_)CH_2_); 7.36 (–Si(CH_3_)_3_).

## 4. Conclusions

The above presented analyses and interpretations permit drawing the following conclusions. Analysis of the research results shows that the loss of Si–H bond conversion takes place directly after introduction of a catalyst portion to the reaction mixture. At that time a jump in temperature takes place leading to almost immediate conversion of Si–H bonds reaching over 90%. Total conversion of Si–H bonds in the system studied was reached after about 55 min of the reaction measured from the moment of the catalyst addition. To sum up a relatively short reaction time and high conversion of the process, reaching 97%, indicate that the system is highly effective. The isolated product was subjected to spectroscopic analysis (^1^H-NMR, ^13^C-NMR, ^29^Si-NMR). A GPC measurement was also performed to analyze the size and molecular weight distribution of the product. In addition, thermal stability tests of the product were carried out. The product structure was confirmed.

As shown by the surface tension, density as well as spectrofluorimetric measurements the critical micelle concentration as well as the surface tension of aqueous solutions of TS-OE12 decrease with increasing temperature. This is probably related to the fact that the degree of hydration of the studied surfactant (TS-OE12) molecules is reduced when they are at the water-air interface and in the micelles.

The partial molar volume, likewise the apparent molar volume of TS-OE12 increase with temperature, which is probably connected with the increase in the average distance between TS-OE12 and water molecules for the surfactant concentrations lower than the critical micelle concentration and between surfactant molecules in the micelles for the surfactant concentrations higher than CMC.

During the micellization process of the studied surfactant, more bonds are broken than formed.

Aggregates of studied surfactant, formed in aqueous solution, could be considered as more or less spherical or ellipsoid ones.

## Figures and Tables

**Table 1:** The values of maximal Gibbs surface excess concentration ($\Gamma_{S}^{\max}$), minimal area of TS-OE12 molecules at the water-air interface ($A_{S}^{\min}$), limiting Gibbs surface excess concentration ($\Gamma_{S}^{\infty }$), limiting area ($A_{S}^{0}$), critical micelle concentration (CMC) at 293 K, 303 K and 313 K.

	Temperature [K]		
	293	303	313
$\Gamma_{S}^{\max}$ × 10^6^ [mol/m^2^] ^a^	2.64	2.60	2.52
$A_{S}^{\min}$ [Ǻ^2^] ^b^	62.90	63.86	65.89
$\Gamma_{S}^{\infty }$ × 10^6^ [mol/m^2^] ^c^	2.69	2.64	2.55
$A_{S}^{0}$ [Ǻ^2^] ^d^	61.80	63.00	65.00
CMC × 10^−4^ [mol/dm^3^] ^e^ (from $\gamma_{LV}$)	3.12	2.53	2.08
CMC × 10^−4^ [mol/dm^3^] ^f^ (from *ρ*)	2.20	2.18	2.15
CMC × 10^−4^ [mol/dm^3^] ^g^ (from *I*_1_/*I*_3_)	4.9	4.07	3.89

^a^ Uncertainty of calculation was ±0.12 mol/m^2^; ^b^ Uncertainty of calculation was ±1.02 Ǻ^2^; ^c^ Uncertainty of calculation was ±0.14 Ǻ^2^; ^d^ Uncertainty of calculation was ±1.04 Ǻ^2^; ^e^ Uncertainty of calculation was ±0.13 × 10^−4^ mol/dm^3^; ^f^ Uncertainty of calculation was ±0.3 × 10^−4^ mol/dm^3^; ^g^ Uncertainty of calculation was ±0.25 × 10^−4^ mol/dm^3^.

**Table 2:** The values of standard Gibbs free energy ($\Delta G_{ads}^{0}$), standard enthalpy ($\Delta H_{ads}^{0}$) and standard entropy ($\Delta S_{ads}^{0}$) of TS-OE12 adsorption as well as standard Gibbs free energy ($\Delta G_{mic}^{0}$), standard enthalpy ($\Delta H_{mic}^{0}$ ) and standard entropy ($\Delta S_{mic}^{0}$) of TS-OE12 micellization at 293 K, 303 K and 313 K.

	Temperature [K]		
	293	303	313
$\Delta G_{ads}^{0}$ [kJ/mol] ^a^	−45.91	−48.3	−50.33
$\Delta H_{ads}^{0}$ [kJ/mol] ^b^	18.78	18.78	18.78
$\Delta S_{ads}^{0}$ [kJ/molK] ^c^	0.22	0.22	0.22
$\Delta G_{mic}^{0}$ [kJ/mol] ^d^	−29.45	−30.97	−32.49
$\Delta H_{mic}^{0}$ [kJ/mol] ^e^	15.12	15.12	15.12
$\Delta S_{mic}^{0}$ [kJ/molK] ^f^	0.15	0.15	0.15

^a–f^ Uncertainty of $\Delta G_{mic}^{0}$, $\Delta H_{mic}^{0}$ and $\Delta S_{mic}^{0}$ calculation was ±0.35 KJ/mol.

## Supplementary Materials

The following are available online, Figure S1: A plot of mole fraction of the area occupied by TS-OE12 at the water-air interface ($X_{S}$) vs. the logarithm of its concentration ($\log C_{S}$) at a temperature equal to 293 K, 303 K and 313 K determined from Equation (4), Figure S2: A plot of mole fraction of the area occupied by TS-OE12 at the water-air interface ($X_{S}$) vs. the logarithm of its concentration ($\log C_{S}$) at a temperature equal to 293 K, 303 K and 313 K determined from Equation (6), Figure S3: A plot of logarithm of mole fraction of the area occupied by TS-OE12 at the water-air interface ($\log X_{S}$) vs. the logarithm of its concentration ($\log C_{S}$) at a temperature equal to 293 K, 303 K and 313 K determined from Equation (6), Figure S4: Values of surface tension ($\gamma_{\mathrm{LV}}$) of aqueous solutions of TS-OE12 (curves 1–3) determined from Equation (7) at 298 K, 303 K and 313 K vs. the logarithm of surfactant concentration ($\log C_{S}$). Figure S5: A plot of the pyrene (*I*_1_/*I*_3_) and (*I*_3_/*I*_1_) ratios vs. the logarithm of TS-OE12 concentration ($\log C_{S}$) at 303 K, Figure S6: A plot of the pyrene (*I*_1_/*I*_3_) and (*I*_3_/*I*_1_) ratios vs. the logarithm of TS-OE12 concentration ($\log C_{S}$) at 313 K.
