# Supplementary material for: Thermodynamic Analysis of Trisiloxane Surfactant Adsorption and Aggregation Processes
Source: Molecules. 2020 Dec 1;25(23):5669. doi: 10.3390/molecules25235669 (PMC7730224; doi:10.3390/molecules25235669)

SUPPLEMENTARY MATERIAL

THERMODYNAMIC ANALYSIS OF TRISILOXANE SURFACTANT ADSORPTION AND AGGREGATION PROCESSES

JOANNA KARASIEWICZ[a*], JOANNA KRAWCZYK[b*]

[a]*Chemistry and Technology of Silicon Compounds, Faculty of Chemistry, Adam Mickiewicz University in Poznań, Uniwersytetu Poznańskiego 8, 61-614 Poznań, Poland*
[b] *Department of Interfacial Phenomena, Institute of Chemical Sciences, Faculty of Chemistry, Maria Curie-Skłodowska University in Lublin, Maria Curie-Skłodowska Sq. 3, 20-031 Lublin, Poland*

*** Corresponding author: e-mail address** j.krawczyk@poczta.umcs.lublin.pl (J. Krawczyk)

and joanka@amu.edu.pl (J. Karasiewicz)

Fig. S1. A plot of mole fraction of the area occupied by TS-OE12 at the water-air interface (X_S) vs. the logarithm of its concentration ($\log C_S$) at a temperature equal to 293 K, 303 K and 313 K determined from Eq. (4).

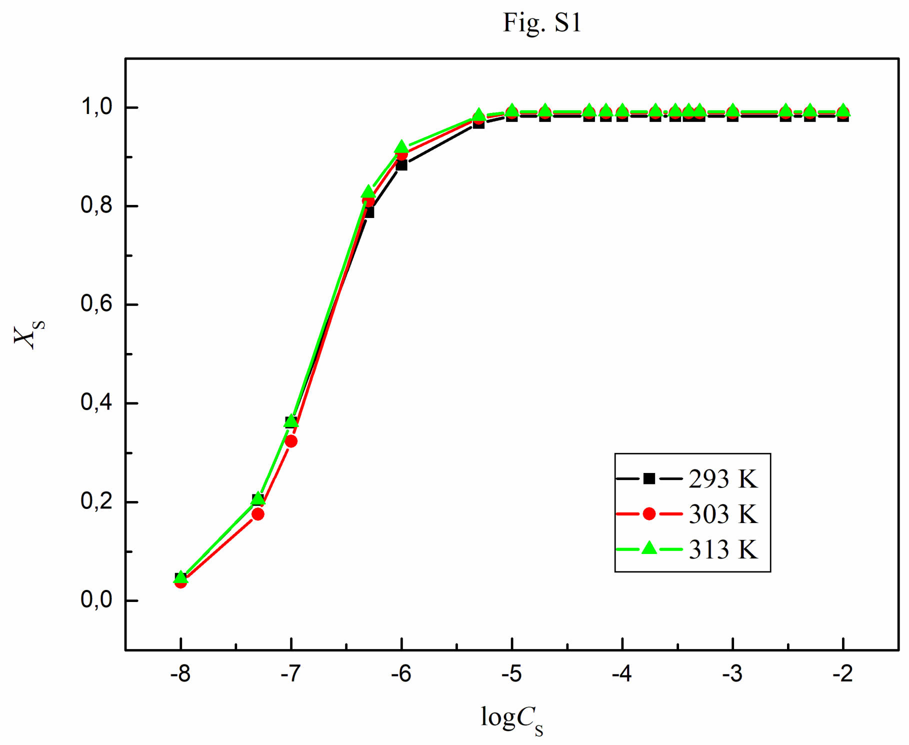

Fig. S2. A plot of mole fraction of the area occupied by TS-OE12 at the water-air interface (X_S) vs. the logarithm of its concentration ($\log C_S$) at a temperature equal to 293 K, 303 K and 313 K determined from Eq. (6).

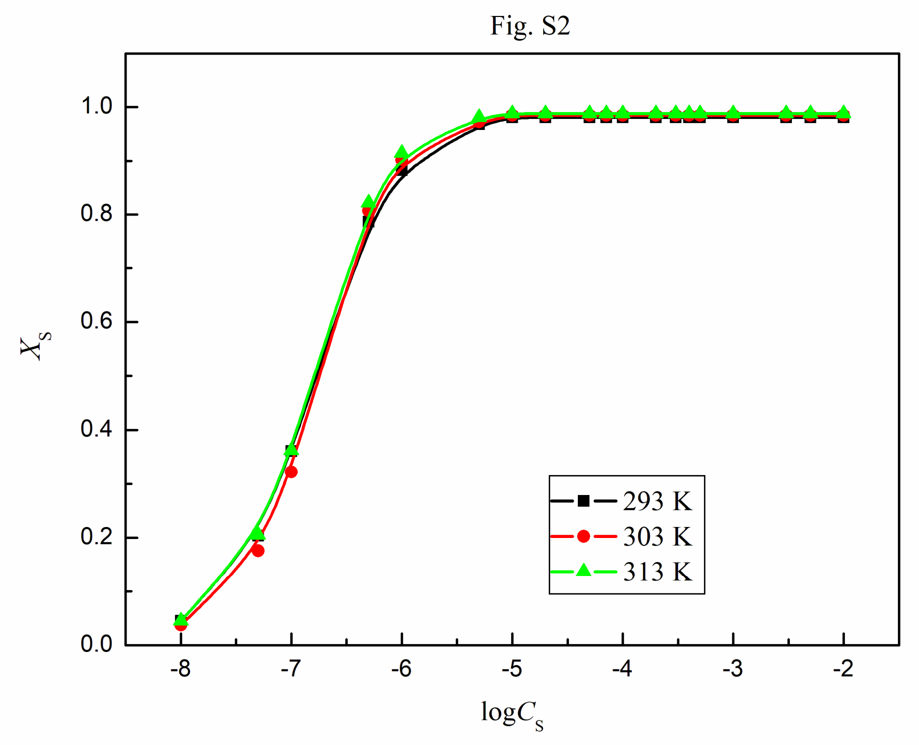

Fig. S3. A plot of logarithm of mole fraction of the area occupied by TS-OE12 at the water-air interface (X_S) vs. the logarithm of its concentration ($\log C_S$) at a temperature equal to 293 K, 303 K and 313 K determined from Eq. (6).

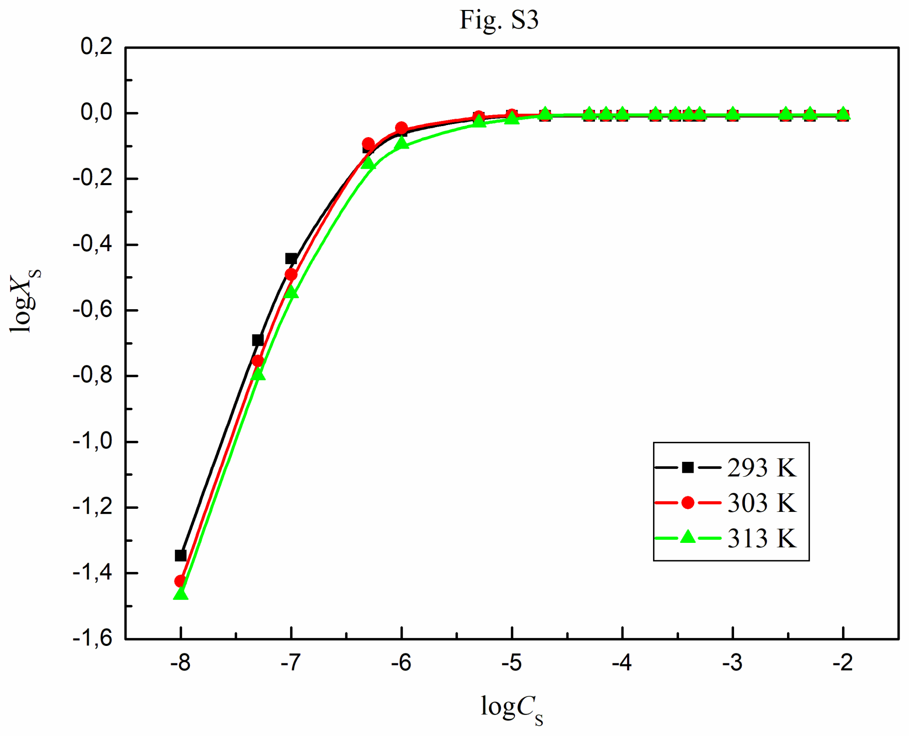

Fig. S4 Values of surface tension (γ_{LV}) of aqueous solutions of TS-OE12 (curves 1–3) determined from Eq. (7) at 298 K, 303 K and 313 K vs. the logarithm of surfactant concentration ($\log C_S$).

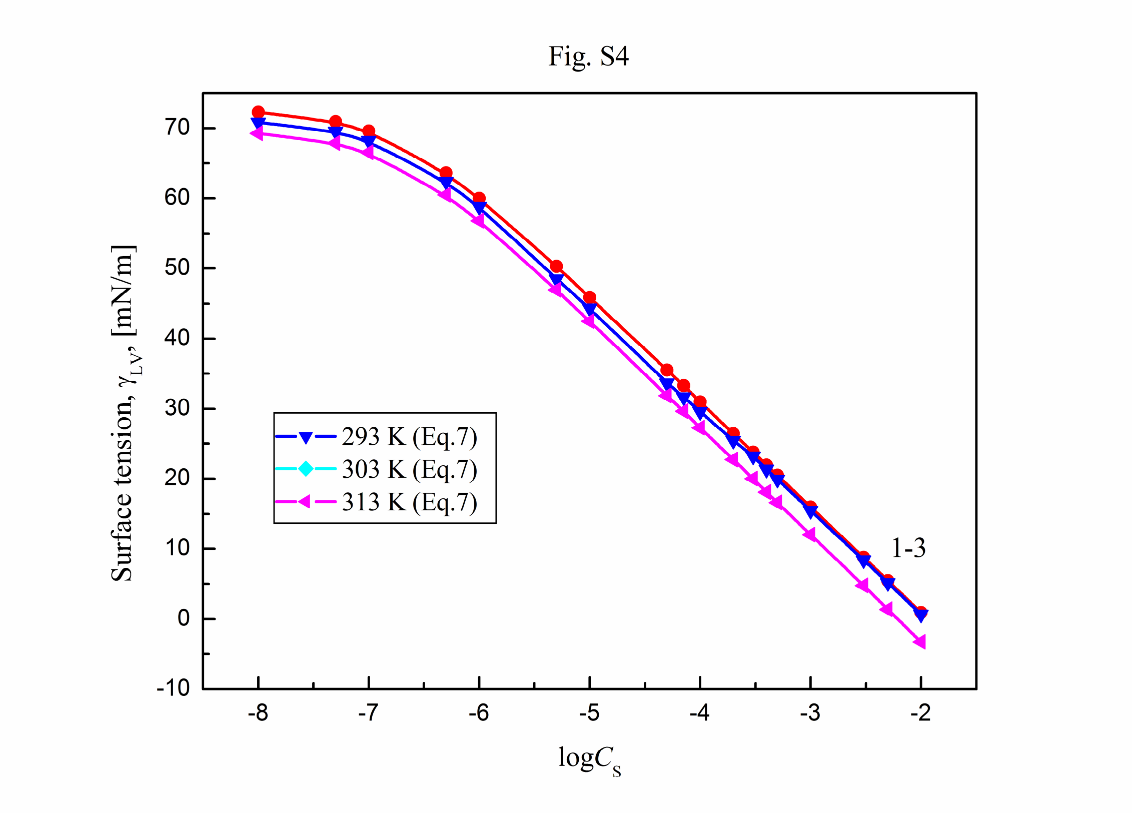

Fig. S5 A plot of the pyrene I_1 / I_3 and I_3 / I_1 ratios vs. the logarithm of TS-OE12 concentration ($\log C_S$) at 303 K.

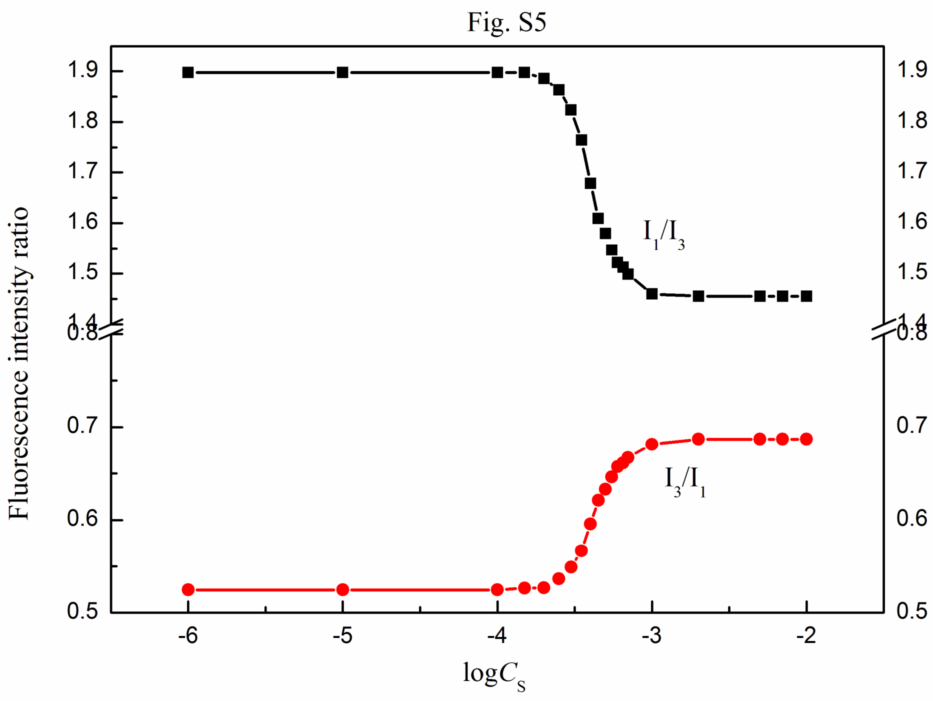

Fig. S6 A plot of the pyrene I_1 / I_3 and I_3 / I_1 ratios vs. the logarithm of TS-OE12 concentration ($\log C_S$) at 313 K.

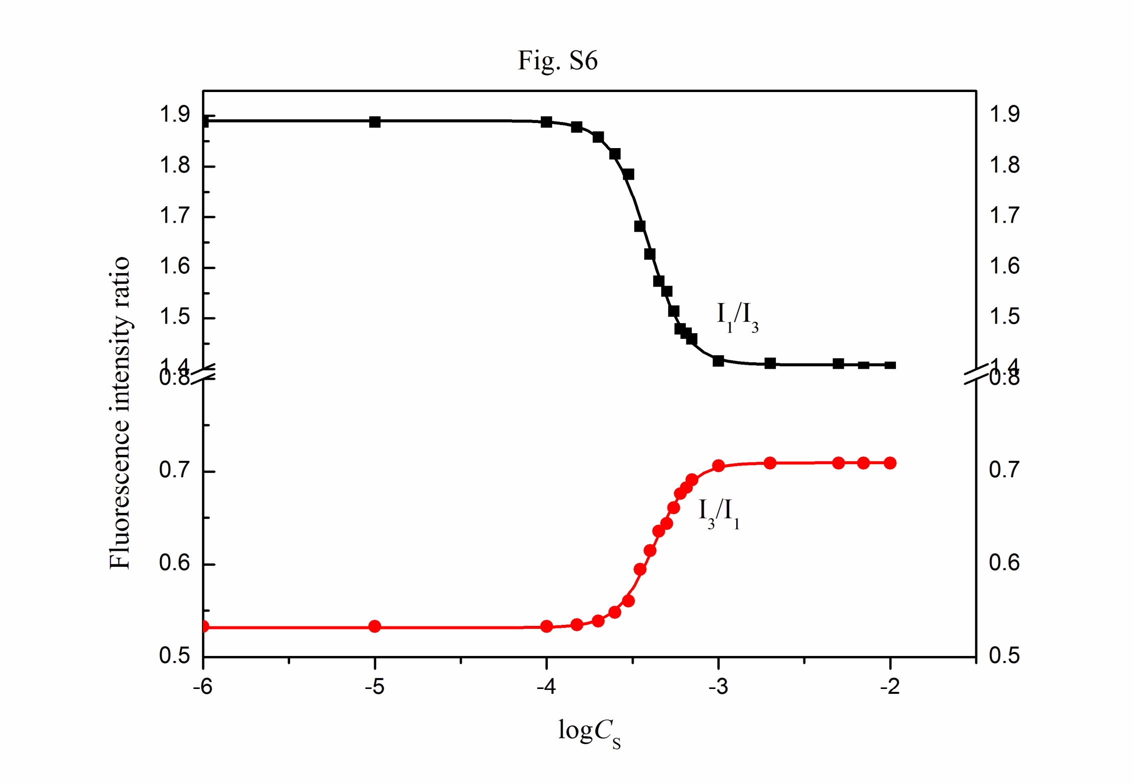

Supplement: Supplementary file 1 [file molecules-25-05669-s001.pdf]
